# Supplementary material for: Deletion of wfs1 Impairs Oligodendrocyte Precursor Cells Dorsal Distribution and Myelination Through the wfs1-hmgcs1 Axis in Zebrafish
Source: Biology (Basel). 2026 Aug 16;15(16):1402. doi: 10.3390/biology15161402 (PMC13510151; doi:10.3390/biology15161402)
Supplement: Supplementary file 1 [file biology-15-01402-s001.zip › File S1-Transcriptome sequencing data and differentially expressed genes (DEGs) in wfs1-- zebrafish at 5 dpf/2.KEGG/wfs1KOvsWT/ALL/src/dre00330.html]

dre00330


- dre:555401

- Up regulated genes

555401(1.88844791660122)

- dre:100005587

- Down regulated genes

100005587\_1(-1.54424530950344)
- dre:100536795

- Down regulated genes

100536795(-1.64304185838866)
- dre:368239

- Up regulated genes

368239(1.14034759711527)
- dre:100006238

- Down regulated genes

100006238\_1(-1.31531604695891)

- dre:100005587

- Down regulated genes

100005587\_1(-1.54424530950344)
- dre:100536795

- Down regulated genes

100536795(-1.64304185838866)
- dre:368239

- Up regulated genes

368239(1.14034759711527)
- dre:100006238

- Down regulated genes

100006238\_1(-1.31531604695891)

- dre:321892

- Up regulated genes

321892\_1(2.16679543930161)

- Down regulated genes

321892(-6.75856243102007)
- dre:327506

- Up regulated genes

327506\_1(5.72916190650829)

- dre:393799

- Up regulated genes

393799(1.39715671800951)

- dre:393799

- Up regulated genes

393799(1.39715671800951)

- dre:405800

- Up regulated genes

405800(1.1459925069877)
- dre:619259

- Up regulated genes

619259(1.74407736881054)

- dre:406688

- Down regulated genes

406688(-1.12341806422973)

Close
